# One‐way endobronchial valves in the management of complex persistent air leaks in a soft tissue sarcoma patient

**DOI:** 10.1111/1759-7714.15064

**Published:** 2023-08-09

**Authors:** Alfonso Fiorelli, Lucia Cannella, Francesca Capasso, Antonio Pizzolorusso, Feranda Picozzi, Gaetana Messina, Giovanni Natale, Edoardo Mercadante, Salvatore Tafuto

**Affiliations:** ^1^ Thoracic Surgery Unit University of Campania Luigi Vanvitelli Caserta Italy; ^2^ S.C. Sarcomi e Tumori Rari, Istituto Nazionale Tumori‐IRCCS‐Fondazione “G. Pascale” Naples Italy; ^3^ Thoracic Surgery, Istituto Nazionale Tumori, “Fondazione G. Pascale”–IRCCS Naples Italy

**Keywords:** endobronchial valve, persistent air leaks, sarcoma, chemotherapy, bronchoscopy

## Abstract

Complex persistent air leak (PAL) is a clinical condition which is difficult to treat. Herein, we report the clinical case of an 18‐year‐old woman with lung and bone metastases due an ultrarare sarcoma: “round cell sarcoma non‐Ewing”. She developed persistent air leaks due to an alveolopleural fistula which developed following two cycles of chemotherapy with doxorubicin. Chest drainage with suction failed to resolve the air leaks, while surgical treatment was unfeasible due to the poor clinical condition of the patient. Thus, she was reviewed for endoscopic treatment with one‐way endobronchial valves. A small valve was sequentially inserted within each segment of the right upper bronchus to occlude the entire upper lobe. Two days after the procedure, resolution of the air leaks were obtained. Chest drainage was removed 5 days later and the patient was discharged. Chemotherapy was resumed. The patient died 7 months later because of disease progression.

## Graphical abstract

A chest computed tomography (CT) scan showed the presence of an alveolopleural fistula within the right side due to the presence of persistent air leaks. After valve insertion within right upper lobe, chest CT scan showed re‐expansion of the lung with obliteration of the pleural cavity.

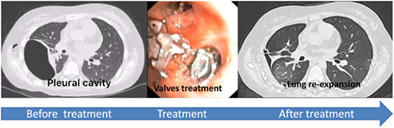

## INTRODUCTION

Complex persistent air leak (PAL) due to alveolopleural fistula (APF) following chemotherapy is a clinical condition which is difficult to treat. It may be associated with significant morbidity and mortality and with reduction of survival due to the delay of treatment for cancer. Thus, an early resolution is desirable.

Herein, we report the successful management of complex PAL due to APF which developed following chemotherapy for soft tissue sarcoma (STS) by endoscopic insertion of one‐way endobronchial valves, designed for the management of emphysema.^1^, ^2^, ^3^

## CASE REPORT

**FIGURE 1:**
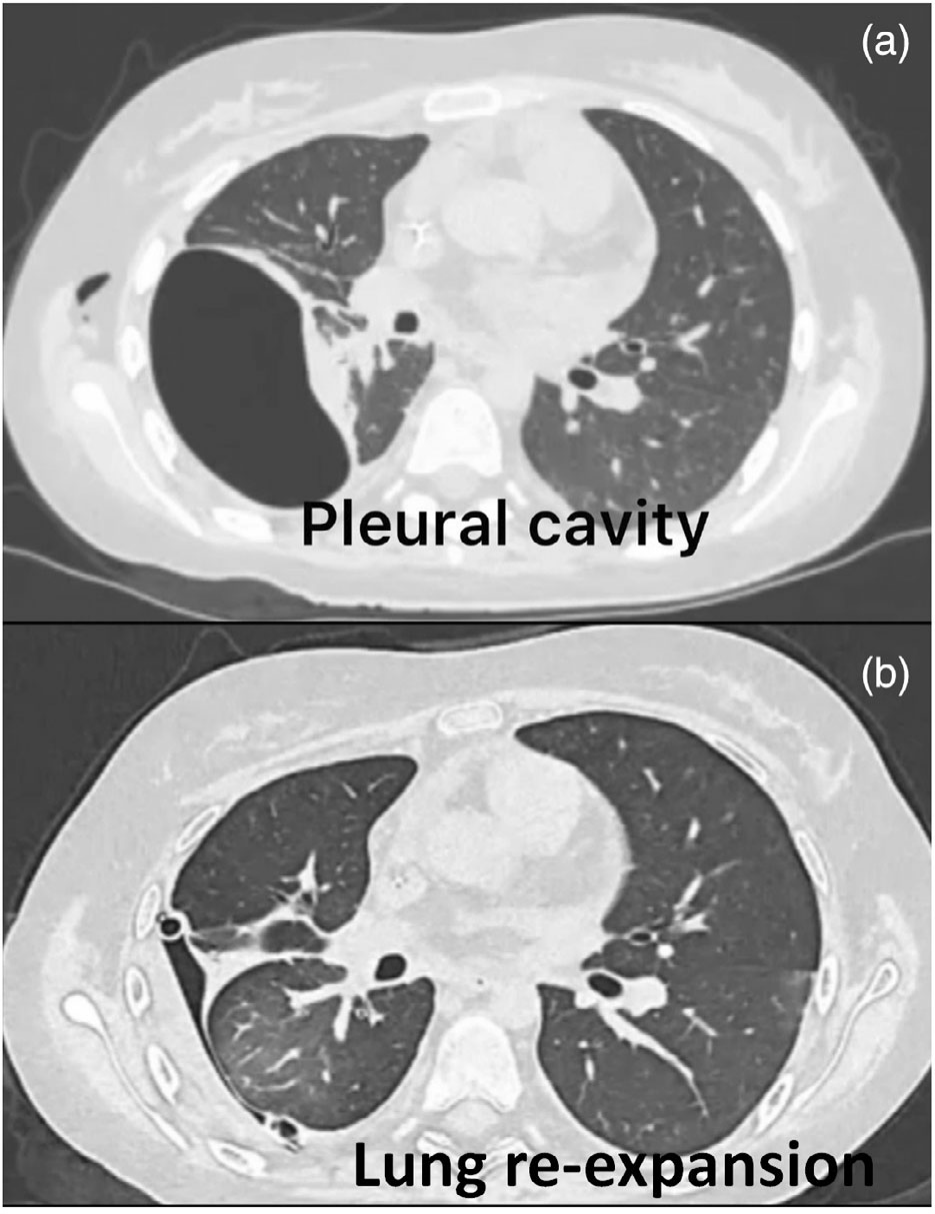
(a) Chest computed tomography (CT) scan revealed a loculated pneumothorax within the right upper lobe due to the presence of persistent air leaks. (b) Chest CT scan performed 2 days after treatment showed re‐expansion of the lung with the resolution of air leaks.

**FIGURE 2:**
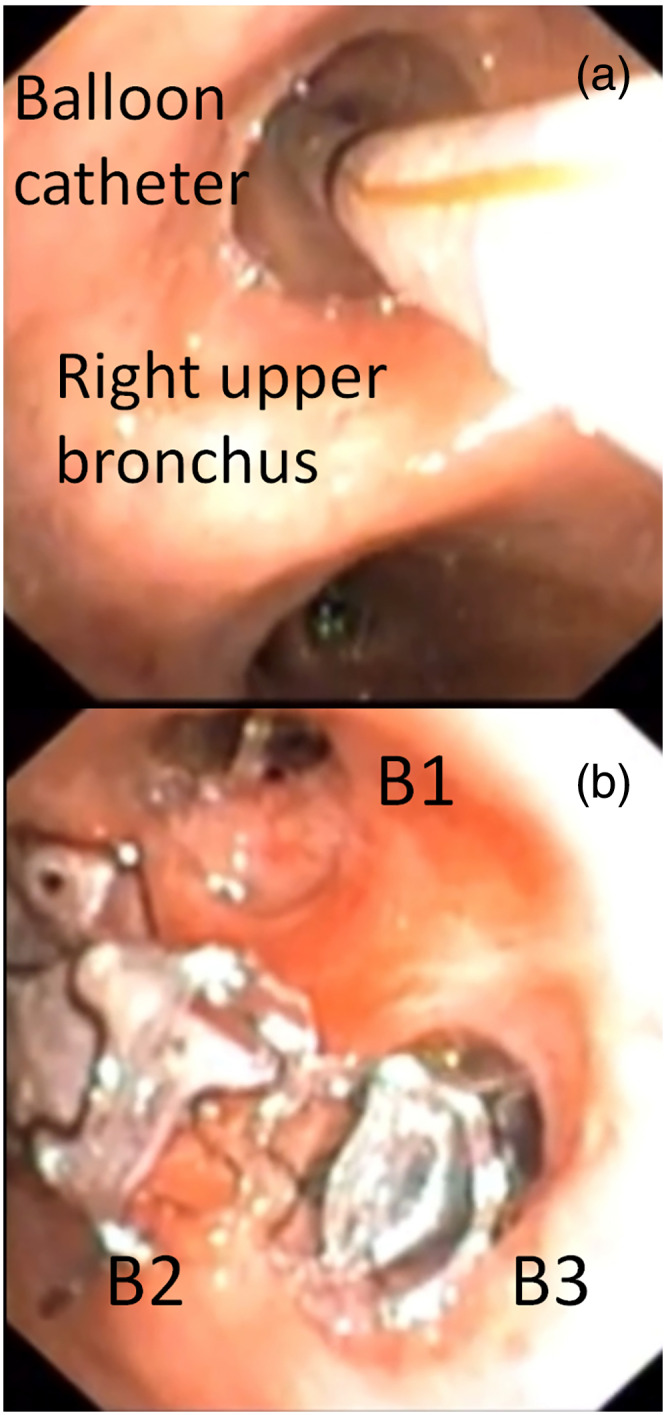
(a) Occlusion of the right upper bronchus with a balloon catheter to find the source of air leaks. (b) Insertion of valves within the segments of the right upper bronchus.

In June 2022, an 18‐year‐old woman underwent treatment with doxorubicin for the management of advanced round cell soft tissue sarcoma with lung and bone metastases. Following two cycles of chemotherapy, probably as a result of tissue response to treatment, the clinical course was complicated by a right‐sided pneumothorax. A chest drain was inserted and connected to suction (−15 cmH_2_0). However, severe air leaks prevented complete re‐expansion of the lung. Chest computed tomography (CT) scan performed 20 days later showed a partial re‐expansion of the lung with the presence of a loculated pneumothorax within the right upper lobe (Figure 1a). Bronchoscopy showed no bronchopleural fistula. Surgical treatment was not indicated due to the patient's poor clinical condition while the chemical pleurodesis through the chest drainage was also unfeasible for the lack of the re‐expansion of the lung. Thus, she was referred to our unit for endoscopic treatment with endobronchial valve (EBV) insertion. The procedure was performed under intravenous sedation with spontaneous breathing, and flexible bronchoscopy. An inflatable balloon catheter was sequentially inserted within the bronchial segments of the right upper bronchus and the air leak rate was assessed through the chest tube 5 min later to identify the culprit segment. A reduction in air leaks was observed after the occlusion of each segment of the right upper lobe, but complete resolution of air leaks was obtained after closure of the entire upper right bronchus (Figure 2a). Thus, a small valve (Zephyr 4.0) was sequentially inserted within each segment of the right upper bronchus to occlude the entire upper lobe (Figure 2b). CT scan performed 2 days after the procedure showed complete expansion of the lung with the resolution of air leaks (Figure 1b). The patient was discharged 5 days later. The procedure is summarized in video S1. Chemotherapy with doxorubicin was resumed in association with ifosfamide to obtain control of the disease. However, the patient died 7 months later because of disease progression.

## DISCUSSION

Complex PAL due to APF following chemotherapy is a detrimental clinical condition that increases morbidity and mortality in already compromised patients and delays treatment for cancer with reduction of survival. The management of this clinical condition is challenging, as in the present case where all standard treatments failed or were unfeasible. The tumor necrosis due to cytotoxic chemotherapy directly induced the formation of APF with consequent severe PAL that did not resolve with the insertion of chest drainage connected to suction.^4^ The American College of Chest Physicians^5^ and British Thoracic Society^6^ recommend surgery as definitive management of PAL due to APF, but our patient was unfit for surgery due to her poor clinical condition. Other authors have reported the successful treatment of PAL by medical pleurodesis via chest drainage.^7^, ^8^ However, in the case reported here, this strategy was unfeasible as the severe air leaks prevented the pleural apposition, that was crucial for the success of pleurodesis. Thus, we reviewed the patient for endoscopic treatment by EBV insertion, designed for the management of emphysema.^1^, ^2^, ^3^ Several authors^9^, ^10^, ^11^ have previously reported the successful treatment of complex PAL following surgical resection with EBV. To the best of our knowledge, our experience is the first to describe PAL due to AFP following chemotherapy. This procedure allowed the patient to continue her therapeutic program and the chemotherapy treatment was also facilitated by the improvement in the patient's performance status which in the following months allowed her to receive chemotherapy in a day hospital rather than in ordinary hospitalization with savings in health expenses. For the age of the patient this procedure certainly improved the quality of life although she was suffering from a disease with a poor prognosis.

Compared to surgical repair, EBV is a minimally invasive treatment that may also be performed in patients in poor clinical condition. Additionally, no chemical pleurodesis is required, reducing the risk of infection of pleural cavity. By contrast, it remains an expensive treatment and should be performed only in selected cases. The key to success in our patient was the identification of the source of the air leaks as the occlusion of culprit bronchus by valves reduced the air leaks and allowed expansion of the lung. CT scan may be helpful in localizing APF but it is mandatary to perform a balloon occlusion test during bronchoscopy to identify the culprit bronchus to treat.^9^, ^10^, ^11^ A Fogarty catheter with an inflatable balloon is systematically inflated from the proximal to the distal airways while the air leak in the drainage system is observed for up to 2–3 min. If occlusion of the segmental bronchus leads to reduction or cessation of air leaks, the valves must be positioned in the occluded segmental bronchus. However, the occlusion of one subsegment may be insufficient to treat the air leak due to the collateral ventilation between lung subsegments, and it is necessary to occlude the entire lobar bronchus, as in the case reported here. The inability to find the culprit bronchus precluded treatment with EBVs. Despite the possibility that valves may be removed after the resolution of air leaks, in our case they were left in place to avoid an additional invasive procedure in a frail patient. The safety of this strategy was supported by other studies,^1^, ^2^, ^3^, ^12^, ^13^ in which valves were placed permanently for emphysema or for other medical conditions.

In conclusion, the endoscopic insertion of EBV is a useful strategy in the armamentarium of physicians for the management of complex PAL, especially when other standard treatments fail or are unfeasible due to the poor clinical condition of the patient. However, it remains an expensive procedure and should be performed only in selected cases when the source of PAL is endoscopically found.

Improving the quality of life in patients with progressive disease with a poor prognosis should be the first goal of therapy. Despite this, quality of life is rarely detected in clinical trials of rare diseases and cost–benefit should be assessed on a patient‐by‐patient basis.

## AUTHOR CONTRIBUTIONS

Alfonso Fiorelli: writing paper and paper revision. Lucia Cannella: writing paper. Francesca Capasso: writing paper. Antonio Pizzolorusso: data collection. Feranda Picozzi: data collection. Gaetana Messina: data collection. Giovanni Natale: writing paper. Edoardo Mercadante: data collection. Salvatore Tafuto: paper revision.

## CONFLICT OF INTEREST STATEMENT

The authors disclose no conflict of interest and no funding for this paper.

## Supporting information


**Video S1.** Video edited the main steps of the procedure as the computer tomography scan before and after the treatment, the endoscopic identification of the culprit bronchus by balloon catheter and the insertion of the endobronchial valves within segments of right upper bronchus.
